# Medical Legislation

**Published:** 1891-09

**Authors:** C. H. Oakes

**Affiliations:** Dighton, Mass.


### MEDICAL LEGISLATION.

### SHOULD THE ALLOPATHIC ADMINISTRATION OF NOXIOUS DRUGS BE PROHIBITED BY LAW?

C. H. Oakes, M. D., Dighton, Mass.

Within a few years medical legislation has become a subject of great interest to our friends of the old school, their society meetings and their journals constantly recurring to it, giving it equal prominence with such subjects as bacteria, Koch, and "paratal-loid."

Medical education has suddenly acquired a degree of importance, to the allopathic mind, that would be truly gratifying to the philanthropist, were it not, like some "revivals" of religion, so extemporaneous in quality.

However, that there is a daily revolution of the earth seems to be now conceded in allopathic circles. They even go so far as to recommend courses of study long since adopted by homœopathic schools. Sometimes, too, their therapeutic searchlight rewards the profession with a "discovery"—a very gem in its way—as, for instance, the fact to them unknown, hitherto, that Aconite in one to three drop doses, and taken at bed-time, is "good for" a coryza. (*Medical Analectic*, January, 1885.) See also in the same journal Professor Bartholow's explanation of the action of Phosphorus, *in very small doses*, in atrophy of the liver, its action being supposed to be due to its antagonism to that organ. (Since Hahnemann's day, what homœopath has not recognized the "antagonism" existing between Phosphorus and the liver, and prescribed accordingly, when the symptoms indicated "Phosphorus in very small doses"?)

Some there are, also, who are waking up to the inutility, and worse, of opiates and of local medicinal applications. It is refreshing to hear from the more advanced among them of the successful use of simple hot water when Morphine had failed to afford relief.

The foregoing are occasional specimens of a somewhat tardy, eleventh-hour progress, and are deserving of a cordial support and a "God-speed." But what of their practice in general, after each "scientific discovery," each "modern improvement," each "revolution in medicine" has had its little day, added its few victims to the grand total of the slain and passed off the stage only to be followed by another experiment—(and pray God it may not reach *their ears!*) another "experiment on the sick"?

For an answer to the above question we will briefly turn to the open pages of old-school literature and accept the story therein contained—whether it be simply one of dissatisfaction and querulous plaint at the paucity of results, or of quasi pride in what "the autopsy revealed."

If it should incidentally appear that in the estimation of the authorities quoted, "Confession is good for the soul," it may be taken as evidence of an innate desire on their part to do good to at least a part of the human economy, and in the manner to them most familiar. The readers of THE HOMŒOPATHIC PHYSICIAN need not fear, however, that I am about to present these confessions in their entirety. A "pocket edition" will suffice.

Beginning with the *armamentarium* of the old school, it will be of interest to hear of its present knowledge, or want of knowledge, of most drugs, the variable strength of different samples of the same drug and the consequent unreliability of the same when used in the sick-room. And it must be decidedly interesting to the public to learn from allopathic sources that there is no fixed standard of strength or action, one sample being found sometimes to differ from another of the same drug in the proportion of one to fourteen.

Bearing upon this point may be cited the words of F. A. Castle, M. D., in that standard and progressive allopathic journal, the *Medical Record:*

"There are very few drugs of which we can certainly specify the constituents in which their therapeutic activity resides; and as for the exact proportion in which these constituents should exist in the crude drug, we know probably less. The reason for this is that no two specimens of the crude drug are alike in this regard, and that we have no reliable means for extracting these constituents for examination which will insure us that the product represents all of their constituents contained by the drug; nor have we the tests which will differentiate between the various constituents of the same plant, some of which may be therapeutically active while others are inert, or have a different action.

"Moreover, it is believed that in plants having several constituents their relative proportions vary in nearly every case, and depend upon the conditions under which the plant grows."

When the full significance of the above quotation is appreciated can any one question the desirability of writing, publishing, and inwardly digesting by our friends of the dominant school an entire volume devoted to the "*Untoward Effects of Drugs*"?

If there still remain those who cherish a lingering belief that some marked progress is being made in the administration of drugs by the so-called "regular" method, the following from the teachers of that method ought to disabuse them of the error. We will listen first to J. Milner Fothergill, author of a *Hand-Book of Treatment*:

"It is eminently desirable that a medical man be generally well informed; but what is to be still more devoutly wished for is that he shall be a skillful practitioner. It is quite possible to be the one without being the other. * * * * The tendency of recent teaching has been rather to produce the first, leaving the second quality to develop itself or to remain in a condition of imperfect evolution, as might fall out. This is not an individual opinion, in which case it would have little weight, but general comment. * * * * Even members of the profession are to be found who assert that the man under whose treatment they would place themselves if seriously ill is the old-fashioned general practitioner. This is a very serious reproach to all our recent advances in scientific medicine; to our modern instruments of precision in diagnosis; and even to our progress in rational therapeutics, with the remedies added to our armamentarium in late years."

Of like character is the testimony of the distinguished author of *A Treatise on the Principles and Practice of Medicine*, Prof.

Austin Flint: "It is worthy of note that our knowledge of the most important remedies has been acquired wholly by experience, without any explanation of their *modus operandi.* * * * * It may perhaps safely be said that the greater success attending the management of diseases now than heretofore is due as much to improvements as regards diet, ventilation, etc., as to the more judicious use of remedial agencies."

And in harmony with the foregoing therapeutic *nihilism* may be quoted *The National Dispensatory*—Stille & Maisch—article Opium, that drug so precious to allopathic physicians the world over—their drug of drugs—*and without which they have confessed themselves unarmed.*

Of this we read, "The attempts to explain the operation of Opium have not been much more satisfactory than in the case of other really efficient medicines. Its local anæsthetic action, and that which its internal use manifests when less than soporific doses are administered, are absolutely unintelligible."

Still more—and this time it is from the report of the One Hundred and Twenty-fourth Annual Meeting of the Medical Society of the State of New Jersey—old enough, certainly, to be respectable, "regular," and reliable. Listen to the words of a vice-president of this venerable body: "While the more intelligent were coming to recognize the fact that health was to be obtained by inhaling the cool and pure air at the sea, on the mountains, by rowing, climbing, etc., rather than by dosing with drugs, yet let him who believed that drugs were going out of date consult the prescription-books of the apothecaries, or the list of drugs imported into this country the past year. There had been over a million dollars' worth imported, the vast majority of which was for the invalid world." The speaker then proceeded to enumerate a few of the deadly drugs imported, giving the amount of each, which in many instances was hundreds of thousands of pounds.

With this startling array before him, the speaker candidly closed with these words *at the one hundred and twenty-fourth annual meeting of his society:* "While drugs had their place, yet it could be safely said that the sanitarian had saved more persons than all the doctors of the last century, Jenner excepted."

Evidently the worthy vice-president was not giving his New York brethren any credit for discovering during the reign of la grippe how to antidote some of their doses by administering—as they stated with a refreshing and child-like simplicity—"whiskey to counteract the depressing influence of the drugs."

With a few words from an address by H. C. Wood, this dreary recital of medical self-condemnation will close.

While lamenting medical ignorance and governmental indifference to the same, he refers to the Conemaugh disaster by way of illustration: "In the presence of the dead of Conemaugh the nation bows in sorrow; but before God I tell you that it is my belief, founded in the largest experience, that if the dead who in the last fifty years have been sacrificed in these United States upon the altar of professional ignorance could this day rise before us, the thousands of Conemaugh would be lost in the multitude; silently, heralded by no roar of flood, mourned by no outburst of national remorse or sorrow, one by one, they have passed over; a never-ending holocaust to governmental imbecility."

It is the blessed privilege of the followers of Hahnemann to "rejoice and be exceeding glad," for whatever their weaknesses they cannot be accused of contributing to the slaughter attending the reckless administration of crude drugs so eloquently portrayed in the foregoing extract.

Can any homœopathic (?) weakling, with these confessions before him, think to increase his usefulness and success by adopting the self-condemned weapons of his allopathic brother, and trying to "practice both ways"? If he has such dreams, let him find his level among his kind—and endeavor to obtain a "higher medical education."

Meanwhile, to the earnest workers among the sick, the question comes home—a cry of suffering from "the invalid world"—*Should not the allopathic administration of noxious drugs be prohibited by law?*

